# 1,5-Dimethyl-4-(1-methyl-3-oxo-3-phenylprop-1-enyl­amino)-2-phenyl-1*H*-pyrazol-3(2*H*)-one

**DOI:** 10.1107/S1600536811021945

**Published:** 2011-06-11

**Authors:** Hualing Zhu, Zhan Wang, Junjie Ren, Miao Zhang, Wei Xu

**Affiliations:** a Department of Basic Science, Tianjin Agriculturial College, Tianjin Jinjing Road No 22, Tianjin, 300384, People’s Republic of China

## Abstract

In the title compound, C_21_H_21_N_3_O_2_, an intra­molecular N—H⋯O inter­action generates an *S*(6) ring, which stablizes the enamine–keto tautomer. The *S*(6) ring makes dihedral angles of 33.07 (7), 56.50 (8) and 38.59 (8)°, respectively, with the benzoyl­acetone benzene ring and the anti­pyrine pyrazole and benzene rings.

## Related literature

For the anti­bacterial activity of Schiff bases, see: Zhang *et al.* (2008 ▶); Li *et al.* (2000 ▶). For general background to anti­pyrine, see: Filho *et al.* (1998 ▶); Bondock *et al.*(2008 ▶). For applications of 4-amino anti­pyrine Schiff bases, see: Meffin *et al.* (1977 ▶); Omar *et al.* (2006 ▶). For Schiff bases derived from aldehyde and 4-amino­anti­pyrine, see: Hay (2007 ▶); Raman *et al.* (2007 ▶). For our previous work on anti­pyrine Schiff bases, see: Zhu *et al.* (2011 ▶). For a related structure, see: Goh *et al.* (2009 ▶).
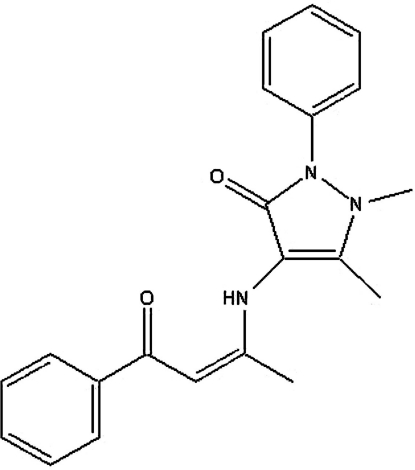


## Experimental

### 

#### Crystal data

C_21_H_21_N_3_O_2_
*M* *_r_* = 347.41
Monoclinic, 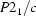

*a* = 9.9418 (12) Å
*b* = 18.456 (3) Å
*c* = 10.1151 (14) Å
β = 104.361 (2)°
*V* = 1798.0 (4) Å^3^
*Z* = 4
Mo *K*α radiation
μ = 0.08 mm^−1^
*T* = 113 K
0.20 × 0.18 × 0.14 mm

#### Data collection

Rigaku Saturn724 CCD diffractometer
Absorption correction: multi-scan (*CrystalClear*; Rigaku, 2001 ▶) *T*_min_ = 0.983, *T*_max_ = 0.988
18576 measured reflections
3160 independent reflections
2910 reflections with *I* > 2σ(*I*)
*R*_int_ = 0.061

#### Refinement

*R*[*F* ^2^ > 2σ(*F* ^2^)] = 0.069
*wR*(*F* ^2^) = 0.180
*S* = 1.11
3160 reflections
242 parameters
1 restraint
H atoms treated by a mixture of independent and constrained refinement
Δρ_max_ = 0.20 e Å^−3^
Δρ_min_ = −0.24 e Å^−3^

### 

Data collection: *CrystalClear* (Rigaku, 2001 ▶); cell refinement: *CrystalClear*; data reduction: *CrystalClear*; program(s) used to solve structure: *SHELXS97* (Sheldrick, 2008 ▶); program(s) used to refine structure: *SHELXL97* (Sheldrick, 2008 ▶); molecular graphics: *SHELXTL* (Sheldrick, 2008 ▶); software used to prepare material for publication: *CrystalStructure* (Rigaku, 2001 ▶).

## Supplementary Material

Crystal structure: contains datablock(s) I, global. DOI: 10.1107/S1600536811021945/jh2294sup1.cif

Structure factors: contains datablock(s) I. DOI: 10.1107/S1600536811021945/jh2294Isup2.hkl

Supplementary material file. DOI: 10.1107/S1600536811021945/jh2294Isup3.cml

Additional supplementary materials:  crystallographic information; 3D view; checkCIF report

## Figures and Tables

**Table 1:** Hydrogen-bond geometry (Å, °)

*D*—H⋯*A*	*D*—H	H⋯*A*	*D*⋯*A*	*D*—H⋯*A*
N3—H3⋯O2	0.90 (1)	1.81 (2)	2.591 (3)	143 (3)

**Fig. 1.**
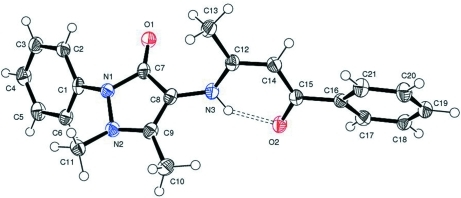
The molecular structure of the title compound with the atom numbering scheme. Displacement ellipsoids are drawn at the 30% probability level. H atoms are presented as a small spheres of arbitrary radii.

## supplementary crystallographic information

### Comment

4-amino antipyrine derivatives have been widely used in the analgesic, anti-bacterial and antitumor field and chemical analysis (Filho *et al.*, 1998; Bondock *et al.*, 2008). While 4-amino antipyrine Schiff bases have showed unique properties and application in the biological, clinical, pharmaceutical and analytical fields (Omar *et al.*, 2006; Meffin *et al.*, 1977). In recent years, more studies concern for the Schiff bases derived from aldehyde and 4-aminoantipyrine (Raman *et al.*, 2007; Hay, 2007), while less concern for the compound derived from ketone and 4-aminoantiprine. In continuation of our studies on antipyrine schiff bases (Zhu *et al.*, 2011), we herein report the crystal structure of the title compound. The molecular structure of the title compound is shown in Fig. 1. An intramolecular N—H···O interaction generates a six- membered ring, producing an S(6) ring (O2 N3 C12 C14 C15), which stablizing the enamine–keto form of the compound. The S(6) ring makes dihedral angles of 33.07 (7)°,56.55 (8)° and 38.59 (8)° with the benzene ring of benzoylacetone, the pyrazole ring and benzene ring of antipyrine,respectively. The bond lengths and angles agree well with those closely related pyrazole structures (Goh *et al.*, 2009).

### Experimental

The title compound was synthesized by refluxing the mixture of benzoylacetone(15*m* mol) and 4-antipyrine (15*m* mol) in ethanol (100 ml) over a steam bath for about 7 h, then the solution was cooled down to room temperature. After seven days, pale yellow block was obtained and dried in air. The product was recrystallized from ethanol which afforded pale yellow and acerate crystals suitable for *X*–ray analysis.

### Refinement

All H atoms were geometrically positioned and treated as riding on their parent atoms, with C—H = 0.93 Å for the aeomatic, 0.96 Å for the methyl H atoms and N—H= 0.90 Å with *U*ĩso~(H)= 1.2 *U*~eq~(Caromatic, N) or, 1.5*U*~eq~(Cmethyl).

### Crystal data

**Table:**

C_21_H_21_N_3_O_2_	*F*(000) = 736
*M**_r_* = 347.41	*D*_x_ = 1.283 Mg m^−^^3^
Monoclinic, *P*2_1_/*c*	Mo *K*α radiation, λ = 0.71073 Å
*a* = 9.9418 (12) Å	Cell parameters from 6064 reflections
*b* = 18.456 (3) Å	θ = 2.1–28.2°
*c* = 10.1151 (14) Å	µ = 0.08 mm^−^^1^
β = 104.361 (2)°	*T* = 113 K
*V* = 1798.0 (4) Å^3^	Prism, colorless
*Z* = 4	0.20 × 0.18 × 0.14 mm

### Data collection

**Table:**

Rigaku Saturn724 CCD diffractometer	3160 independent reflections
Radiation source: rotating anode	2910 reflections with *I* > 2σ(*I*)
multilayer	*R*_int_ = 0.061
Detector resolution: 14.22 pixels mm^-1^	θ_max_ = 25.0°, θ_min_ = 2.1°
ω and φ scans	*h* = −11→11
Absorption correction: multi-scan (*CrystalClear*; Rigaku, 2001)	*k* = −21→21
*T*_min_ = 0.983, *T*_max_ = 0.988	*l* = −12→12
18576 measured reflections	

### Refinement

**Table:**

Refinement on *F*^2^	Primary atom site location: structure-invariant direct methods
Least-squares matrix: full	Secondary atom site location: difference Fourier map
*R*[*F*^2^ > 2σ(*F*^2^)] = 0.069	Hydrogen site location: inferred from neighbouring sites
*wR*(*F*^2^) = 0.180	H atoms treated by a mixture of independent and constrained refinement
*S* = 1.11	*w* = 1/[σ^2^(*F*_o_^2^) + (0.0849*P*)^2^ + 1.0899*P*] where *P* = (*F*_o_^2^ + 2*F*_c_^2^)/3
3160 reflections	(Δ/σ)_max_ < 0.001
242 parameters	Δρ_max_ = 0.20 e Å^−^^3^
1 restraint	Δρ_min_ = −0.24 e Å^−^^3^

### Special details

**Table:**

Geometry. All e.s.d.'s (except the e.s.d. in the dihedral angle between two l.s. planes) are estimated using the full covariance matrix. The cell e.s.d.'s are taken into account individually in the estimation of e.s.d.'s in distances, angles and torsion angles; correlations between e.s.d.'s in cell parameters are only used when they are defined by crystal symmetry. An approximate (isotropic) treatment of cell e.s.d.'s is used for estimating e.s.d.'s involving l.s. planes.
Refinement. Refinement of *F*^2^ against ALL reflections. The weighted *R*-factor *wR* and goodness of fit *S* are based on *F*^2^, conventional *R*-factors *R* are based on *F*, with *F* set to zero for negative *F*^2^. The threshold expression of *F*^2^ > σ(*F*^2^) is used only for calculating *R*-factors(gt) *etc*. and is not relevant to the choice of reflections for refinement. *R*-factors based on *F*^2^ are statistically about twice as large as those based on *F*, and *R*- factors based on ALL data will be even larger.

### Fractional atomic coordinates and isotropic or equivalent isotropic displacement parameters (Å^2^)

**Table:**

	*x*	*y*	*z*	*U*_iso_*/*U*_eq_	
O1	0.72095 (18)	1.08766 (10)	0.41989 (19)	0.0284 (5)	
O2	0.83461 (19)	0.93727 (10)	0.00431 (19)	0.0291 (5)	
N1	0.4862 (2)	1.05874 (11)	0.3514 (2)	0.0249 (5)	
N2	0.4038 (2)	1.01384 (12)	0.2496 (2)	0.0263 (5)	
N3	0.7420 (2)	0.96481 (12)	0.2169 (2)	0.0268 (5)	
C1	0.4275 (3)	1.12584 (14)	0.3815 (3)	0.0248 (6)	
C2	0.4802 (3)	1.15799 (15)	0.5078 (3)	0.0287 (6)	
H2	0.5552	1.1362	0.5722	0.034*	
C3	0.4225 (3)	1.22220 (16)	0.5392 (3)	0.0337 (7)	
H3A	0.4590	1.2448	0.6250	0.040*	
C4	0.3118 (3)	1.25348 (15)	0.4457 (3)	0.0352 (7)	
H4	0.2719	1.2972	0.4679	0.042*	
C5	0.2596 (3)	1.22104 (15)	0.3202 (3)	0.0336 (7)	
H5	0.1835	1.2426	0.2565	0.040*	
C6	0.3173 (3)	1.15733 (14)	0.2864 (3)	0.0278 (6)	
H6	0.2822	1.1355	0.1996	0.033*	
C7	0.6269 (3)	1.04979 (14)	0.3528 (3)	0.0250 (6)	
C8	0.6267 (3)	0.99043 (14)	0.2596 (3)	0.0258 (6)	
C9	0.4928 (3)	0.97008 (14)	0.2037 (3)	0.0272 (6)	
C10	0.4406 (3)	0.91013 (15)	0.1055 (3)	0.0344 (7)	
H10A	0.4021	0.8716	0.1517	0.052*	
H10B	0.5174	0.8907	0.0713	0.052*	
H10C	0.3681	0.9287	0.0289	0.052*	
C11	0.2724 (3)	0.98719 (15)	0.2733 (3)	0.0284 (6)	
H11A	0.2200	0.9615	0.1919	0.043*	
H11B	0.2176	1.0282	0.2923	0.043*	
H11C	0.2920	0.9540	0.3514	0.043*	
C12	0.8539 (3)	0.92975 (14)	0.2913 (3)	0.0251 (6)	
C13	0.8703 (3)	0.92201 (16)	0.4421 (3)	0.0329 (7)	
H13A	0.8720	0.9701	0.4834	0.049*	
H13B	0.9573	0.8967	0.4827	0.049*	
H13C	0.7921	0.8942	0.4586	0.049*	
C14	0.9500 (3)	0.90044 (14)	0.2283 (3)	0.0260 (6)	
H14	1.0305	0.8779	0.2833	0.031*	
C15	0.9329 (3)	0.90276 (13)	0.0845 (3)	0.0241 (6)	
C16	1.0308 (3)	0.86266 (13)	0.0208 (3)	0.0231 (6)	
C17	1.0587 (3)	0.88942 (14)	−0.0988 (3)	0.0276 (6)	
H17	1.0171	0.9334	−0.1373	0.033*	
C18	1.1466 (3)	0.85217 (15)	−0.1615 (3)	0.0307 (6)	
H18	1.1660	0.8708	−0.2423	0.037*	
C19	1.2063 (3)	0.78773 (15)	−0.1065 (3)	0.0315 (7)	
H19	1.2671	0.7625	−0.1495	0.038*	
C20	1.1783 (3)	0.75975 (15)	0.0104 (3)	0.0340 (7)	
H20	1.2187	0.7151	0.0470	0.041*	
C21	1.0911 (3)	0.79702 (15)	0.0743 (3)	0.0302 (6)	
H21	1.0720	0.7779	0.1549	0.036*	
H3	0.739 (3)	0.9643 (18)	0.1269 (13)	0.046 (9)*	

### Atomic displacement parameters (Å^2^)

**Table:**

	*U*^11^	*U*^22^	*U*^33^	*U*^12^	*U*^13^	*U*^23^
O1	0.0287 (10)	0.0262 (10)	0.0321 (11)	−0.0030 (8)	0.0107 (8)	−0.0034 (8)
O2	0.0313 (10)	0.0288 (10)	0.0290 (10)	0.0064 (8)	0.0107 (8)	0.0013 (8)
N1	0.0244 (11)	0.0230 (11)	0.0274 (12)	0.0019 (9)	0.0068 (9)	−0.0039 (9)
N2	0.0253 (11)	0.0255 (12)	0.0288 (12)	0.0008 (9)	0.0078 (9)	−0.0028 (9)
N3	0.0276 (12)	0.0285 (12)	0.0265 (12)	0.0038 (10)	0.0105 (10)	0.0011 (10)
C1	0.0270 (14)	0.0192 (13)	0.0326 (15)	0.0021 (10)	0.0158 (12)	0.0006 (11)
C2	0.0259 (14)	0.0302 (15)	0.0309 (15)	0.0011 (11)	0.0087 (12)	−0.0048 (12)
C3	0.0321 (15)	0.0335 (16)	0.0380 (17)	−0.0029 (12)	0.0136 (13)	−0.0089 (13)
C4	0.0361 (17)	0.0240 (14)	0.0487 (19)	0.0034 (12)	0.0167 (14)	−0.0051 (13)
C5	0.0345 (16)	0.0280 (15)	0.0411 (17)	0.0083 (12)	0.0146 (13)	0.0072 (13)
C6	0.0318 (15)	0.0261 (14)	0.0276 (14)	0.0019 (11)	0.0113 (12)	0.0021 (11)
C7	0.0261 (14)	0.0251 (14)	0.0269 (14)	0.0021 (11)	0.0123 (11)	0.0038 (11)
C8	0.0320 (15)	0.0248 (14)	0.0233 (14)	0.0019 (11)	0.0123 (12)	0.0020 (11)
C9	0.0322 (15)	0.0243 (14)	0.0258 (14)	0.0045 (11)	0.0084 (12)	0.0010 (11)
C10	0.0371 (16)	0.0311 (16)	0.0349 (16)	−0.0001 (12)	0.0085 (13)	−0.0100 (13)
C11	0.0237 (14)	0.0307 (15)	0.0317 (15)	−0.0005 (11)	0.0086 (11)	0.0029 (12)
C12	0.0269 (14)	0.0210 (13)	0.0292 (14)	−0.0009 (11)	0.0102 (11)	−0.0022 (11)
C13	0.0348 (16)	0.0391 (16)	0.0267 (15)	0.0043 (13)	0.0112 (13)	0.0003 (12)
C14	0.0247 (14)	0.0277 (14)	0.0260 (14)	0.0011 (11)	0.0068 (11)	0.0030 (11)
C15	0.0237 (13)	0.0198 (13)	0.0304 (14)	−0.0016 (10)	0.0095 (11)	−0.0011 (11)
C16	0.0222 (13)	0.0220 (13)	0.0249 (14)	0.0005 (10)	0.0053 (10)	−0.0022 (10)
C17	0.0272 (14)	0.0243 (14)	0.0306 (15)	−0.0014 (11)	0.0058 (11)	−0.0007 (11)
C18	0.0285 (15)	0.0377 (16)	0.0282 (15)	−0.0047 (12)	0.0116 (12)	−0.0060 (12)
C19	0.0275 (15)	0.0324 (15)	0.0352 (16)	0.0028 (12)	0.0089 (12)	−0.0087 (13)
C20	0.0344 (16)	0.0284 (15)	0.0404 (17)	0.0090 (12)	0.0117 (13)	0.0008 (13)
C21	0.0350 (15)	0.0258 (14)	0.0326 (15)	0.0028 (12)	0.0135 (12)	0.0031 (12)

### Geometric parameters (Å, °)

**Table:**

O1—C7	1.228 (3)	C10—H10B	0.9800
O2—C15	1.275 (3)	C10—H10C	0.9800
N1—C7	1.405 (3)	C11—H11A	0.9800
N1—N2	1.414 (3)	C11—H11B	0.9800
N1—C1	1.434 (3)	C11—H11C	0.9800
N2—C9	1.362 (3)	C12—C14	1.383 (4)
N2—C11	1.470 (3)	C12—C13	1.500 (4)
N3—C12	1.344 (3)	C13—H13A	0.9800
N3—C8	1.404 (3)	C13—H13B	0.9800
N3—H3	0.903 (10)	C13—H13C	0.9800
C1—C2	1.387 (4)	C14—C15	1.423 (4)
C1—C6	1.394 (4)	C14—H14	0.9500
C2—C3	1.387 (4)	C15—C16	1.490 (3)
C2—H2	0.9500	C16—C17	1.397 (4)
C3—C4	1.387 (4)	C16—C21	1.399 (4)
C3—H3A	0.9500	C17—C18	1.383 (4)
C4—C5	1.384 (4)	C17—H17	0.9500
C4—H4	0.9500	C18—C19	1.383 (4)
C5—C6	1.388 (4)	C18—H18	0.9500
C5—H5	0.9500	C19—C20	1.380 (4)
C6—H6	0.9500	C19—H19	0.9500
C7—C8	1.445 (4)	C20—C21	1.386 (4)
C8—C9	1.364 (4)	C20—H20	0.9500
C9—C10	1.492 (4)	C21—H21	0.9500
C10—H10A	0.9800		
			
C7—N1—N2	109.5 (2)	H10B—C10—H10C	109.5
C7—N1—C1	123.8 (2)	N2—C11—H11A	109.5
N2—N1—C1	117.9 (2)	N2—C11—H11B	109.5
C9—N2—N1	106.7 (2)	H11A—C11—H11B	109.5
C9—N2—C11	122.4 (2)	N2—C11—H11C	109.5
N1—N2—C11	117.1 (2)	H11A—C11—H11C	109.5
C12—N3—C8	128.0 (2)	H11B—C11—H11C	109.5
C12—N3—H3	112 (2)	N3—C12—C14	120.0 (2)
C8—N3—H3	119 (2)	N3—C12—C13	118.8 (2)
C2—C1—C6	120.7 (2)	C14—C12—C13	121.2 (2)
C2—C1—N1	119.0 (2)	C12—C13—H13A	109.5
C6—C1—N1	120.3 (2)	C12—C13—H13B	109.5
C1—C2—C3	119.6 (3)	H13A—C13—H13B	109.5
C1—C2—H2	120.2	C12—C13—H13C	109.5
C3—C2—H2	120.2	H13A—C13—H13C	109.5
C4—C3—C2	120.2 (3)	H13B—C13—H13C	109.5
C4—C3—H3A	119.9	C12—C14—C15	122.6 (2)
C2—C3—H3A	119.9	C12—C14—H14	118.7
C5—C4—C3	120.0 (3)	C15—C14—H14	118.7
C5—C4—H4	120.0	O2—C15—C14	122.9 (2)
C3—C4—H4	120.0	O2—C15—C16	116.9 (2)
C4—C5—C6	120.6 (3)	C14—C15—C16	120.1 (2)
C4—C5—H5	119.7	C17—C16—C21	118.9 (2)
C6—C5—H5	119.7	C17—C16—C15	119.3 (2)
C5—C6—C1	119.0 (3)	C21—C16—C15	121.7 (2)
C5—C6—H6	120.5	C18—C17—C16	120.3 (3)
C1—C6—H6	120.5	C18—C17—H17	119.8
O1—C7—N1	124.0 (2)	C16—C17—H17	119.8
O1—C7—C8	132.0 (2)	C19—C18—C17	120.0 (3)
N1—C7—C8	104.0 (2)	C19—C18—H18	120.0
C9—C8—N3	124.6 (2)	C17—C18—H18	120.0
C9—C8—C7	108.8 (2)	C20—C19—C18	120.6 (3)
N3—C8—C7	126.0 (2)	C20—C19—H19	119.7
N2—C9—C8	110.2 (2)	C18—C19—H19	119.7
N2—C9—C10	121.3 (2)	C19—C20—C21	119.7 (3)
C8—C9—C10	128.5 (2)	C19—C20—H20	120.1
C9—C10—H10A	109.5	C21—C20—H20	120.1
C9—C10—H10B	109.5	C20—C21—C16	120.5 (3)
H10A—C10—H10B	109.5	C20—C21—H21	119.8
C9—C10—H10C	109.5	C16—C21—H21	119.8
H10A—C10—H10C	109.5		
			
C7—N1—N2—C9	8.9 (3)	N1—N2—C9—C8	−6.8 (3)
C1—N1—N2—C9	157.7 (2)	C11—N2—C9—C8	−145.8 (2)
C7—N1—N2—C11	150.4 (2)	N1—N2—C9—C10	173.5 (2)
C1—N1—N2—C11	−60.8 (3)	C11—N2—C9—C10	34.6 (4)
C7—N1—C1—C2	−58.8 (3)	N3—C8—C9—N2	−168.8 (2)
N2—N1—C1—C2	157.1 (2)	C7—C8—C9—N2	2.3 (3)
C7—N1—C1—C6	122.5 (3)	N3—C8—C9—C10	10.8 (5)
N2—N1—C1—C6	−21.6 (3)	C7—C8—C9—C10	−178.0 (3)
C6—C1—C2—C3	−0.1 (4)	C8—N3—C12—C14	171.9 (3)
N1—C1—C2—C3	−178.8 (2)	C8—N3—C12—C13	−6.8 (4)
C1—C2—C3—C4	0.8 (4)	N3—C12—C14—C15	−2.9 (4)
C2—C3—C4—C5	−0.7 (4)	C13—C12—C14—C15	175.8 (2)
C3—C4—C5—C6	−0.2 (4)	C12—C14—C15—O2	6.3 (4)
C4—C5—C6—C1	0.9 (4)	C12—C14—C15—C16	−172.6 (2)
C2—C1—C6—C5	−0.8 (4)	O2—C15—C16—C17	31.3 (3)
N1—C1—C6—C5	177.9 (2)	C14—C15—C16—C17	−149.8 (2)
N2—N1—C7—O1	171.5 (2)	O2—C15—C16—C21	−145.8 (3)
C1—N1—C7—O1	24.9 (4)	C14—C15—C16—C21	33.1 (4)
N2—N1—C7—C8	−7.3 (3)	C21—C16—C17—C18	−1.2 (4)
C1—N1—C7—C8	−153.9 (2)	C15—C16—C17—C18	−178.4 (2)
C12—N3—C8—C9	−121.4 (3)	C16—C17—C18—C19	0.6 (4)
C12—N3—C8—C7	69.0 (4)	C17—C18—C19—C20	0.4 (4)
O1—C7—C8—C9	−175.5 (3)	C18—C19—C20—C21	−0.8 (4)
N1—C7—C8—C9	3.1 (3)	C19—C20—C21—C16	0.2 (4)
O1—C7—C8—N3	−4.5 (5)	C17—C16—C21—C20	0.8 (4)
N1—C7—C8—N3	174.1 (2)	C15—C16—C21—C20	177.9 (3)

### Hydrogen-bond geometry (Å, °)

**Table:**

*D*—H···*A*	*D*—H	H···*A*	*D*···*A*	*D*—H···*A*
N3—H3···O2	0.90 (1)	1.81 (2)	2.591 (3)	143 (3)
